# Exploring gender stereotypes and norms among peri-urban very young adolescents in Zimbabwe using participatory and qualitative approaches

**DOI:** 10.1371/journal.pgph.0003845

**Published:** 2025-05-29

**Authors:** Owen Nyamwanza, Tariro S. Bikwayi, Tariro Chinozvina, Leviticus Makoni, Farai Muronzi, Maxwell Changombe, Angela Obasi, Talent Makoni, Sinokuthemba Xaba, Owen Mugurungi, James R. Hargreaves, Frances M. Cowan, Webster Mavhu

**Affiliations:** 1 Centre for Sexual Health and HIV/AIDS Research (CeSHHAR), Harare, Zimbabwe; 2 Restless Development, Harare, Zimbabwe; 3 Department of International Public Health, Liverpool School of Tropical Medicine, Liverpool, United Kingdom; 4 AIDS and TB Unit, Ministry of Health and Child Care, Harare, Zimbabwe; 5 Department of Public Health, Environments and Society, Faculty of Public Health and Policy, London School of Hygiene & Tropical Medicine, London, United Kingdom; PLOS: Public Library of Science, UNITED STATES OF AMERICA

## Abstract

Gender stereotypes and norms shape very young adolescents’ (VYAs, 10–14 years old) behaviours, including in relation to sexual and reproductive health (SRH). This formative study sought to determine and prioritise the stereotypes and norms to be targeted as part of work to co-develop a gender-transformative intervention for VYAs in Zimbabwe to promote positive masculinities and SRH. In 2023, we collected data from VYAs, using participatory workshops encompassing various activities. We also held focus group discussions with older adolescents and parents/guardians, and individual interviews with community influencers. We used interpretive thematic analysis to generate themes across data. We later presented research findings to diverse stakeholders to explore how the findings might influence the design of our gender-transformative intervention. Gender stereotypes emerged in relation to sexual behaviour and SRH norms. Both boys and girls seemed to condone boys’ multiple, concurrent relationships. Boys were deemed to be unable to control their sexual urges. Menstrual stigma, myths and misconceptions were pervasive. Stereotypes were also evident in beliefs and norms around resource and task allocation. For example, both boys and girls concurred that given limited resources, educating a boy child should be prioritised even when a girl sibling is performing better academically. Stereotypes relating to labour distribution were also evident. Daily activity charts suggested longer working hours for girls. Differential attitudes towards drug and substance use among boys and girls were driven by underlying masculine norms. Of note, adolescents disapproved of some of these norms, pointing to an opportunity to shift them. Stakeholders highlighted the need for our planned intervention to focus on the wider community, in addition to VYAs themselves. The formative research enabled us to identify key gender stereotypes and norms, information which is critical for informing the planned gender-transformative intervention. Although deeply seated, these stereotypes are not insurmountable, particularly among VYAs.

## Introduction

Globally, there are ∼1.3 billion adolescents of whom half are very young adolescents (VYAs, 10–14 years old) [1]. In Africa, the number of young people will double in the next 30 years [2]. The World Bank predicts that Africa’s ability to benefit from the projected population growth depends on the health and wellbeing of today’s adolescents [2]. Adolescence has been characterised as ‘a special time for gender’ [3]. During this period, both gender expression and gendered behaviour develop, as social and gender norms are established, whilst others are simultaneously revised or discarded [4]. These gender stereotypical traits are often internalised and reinforced through prescriptive (i.e., what boys/men or girls/women should be/are allowed to be or do) and proscriptive (i.e., what they should not be/do not have to be or do) social norms [5], which persist into adulthood, and impact future health behaviours and outcomes.

Working with boys or men, in addition to girls or women, through gender-transformative programming that challenges gender inequalities and harmful norms related to masculinities, is recognised as important for improving sexual and reproductive health and rights (SRHR) for all [6]. Whereas some SRHR initiatives include boys and men, few embed a gender-transformative approach. Of note, even where programmes have focused on adolescents, these tend to be older adolescents (15–19 years), when change is likely to be more difficult to achieve [7]. A much smaller number have addressed issues with VYA boys (aged 10–14 years) [7–9], where there is a clear opportunity for co-developing and evaluating interventions with potential to be transformative.

The Global Early Adolescent Study - a longitudinal, multi-country initiative - is exploring VYAs’ perceptions of the gender norms that regulate their behaviour, how they form their own beliefs about gender, and how these beliefs align with social norms in their communities, including in four African countries: Democratic Republic of Congo, Kenya, Malawi and South Africa [10]. An important observation has been that, as with other population groups, intervention effects do differ by context, and results can be highly contextual, even for settings generally considered ‘similar’ [11]. Indeed, there is increased recognition that African masculinities are produced in unique and varying contexts of intersections (including class, ethnicity, sexuality) [12]. This points to the need for context-specific, bespoke initiatives.

Our work in Zimbabwe over the past decades has highlighted how gender stereotypes and norms shape older adolescents and young people’s behaviours, including in relation to SRH [13–17]. In this setting, patriarchal structures confer power on men to control resources and dominate women, leading to social and gender norms that are favourable to the former and punitive to the latter [18,19]. There are several other linked norms related to control of female sexuality, fertility and childbearing, and family planning use, which either facilitate or impede healthy SRH behaviours and service seeking [19]. For example, females who either purchase condoms or suggest condom use are considered ‘loose’ and therefore, both unfit and unsuitable for marriage [13,15,16]. Such negative “sanctions” contribute to adolescent girls’ poorer SRH outcomes.

In response to these inequities, we are conducting research to co-develop and evaluate a peer-delivered gender-transformative intervention for VYAs in Zimbabwe to promote positive masculinities (i.e., positive beliefs, practices associated with being a man) [12] and SRH among especially boys. The goal is to develop an effective model for scale-up to influence locally normative constructs of positive masculinities.

Here, we present findings from a formative study to determine and prioritise the gender stereotypes and norms to be targeted.

## Methods

### Sites and participants selection

We recruited study participants in four impoverished peri-urban Harare suburbs: Churu Farm, Epworth, Hopley and Ushewekunze, where our implementing partner, a youth-focused organisation, has been delivering SRH programmes to in- and out-of-school youths. The four suburbs are relatively recent, and home to the burgeoning low-income urban populations. Health, educational and other social amenities are poorly developed and often overstretched. Unemployment levels are unacceptably high, with men and women subsisting on menial jobs (e.g., vending, brick moulding, waste picking). These settlements are also characterised by high levels of crime, prostitution, violence and substance use, vices which likely influence adolescent norms [20].

In this formative study, we included VYAs (10–14 years-old), older adolescents (15–19 years-old), parents/guardians and key informants including teachers, traditional/religious leaders, community influencers and community-based organisation representatives. Our implementing partner assisted with participant recruitment. Purposive sampling ensured a balanced representation by participant age (e.g., 10–12 vs 13–14 year-olds), sex and location.

### Data collection, processing and analysis

We collected data using participatory workshops (n = 4 workshops) with VYAs. To triangulate participatory workshops findings, we conducted single-sex focus group discussions (FGDs) with older adolescents (n = 4 FGDs) and with parents/guardians (n = 2 FGDs). In addition, we conducted in-depth interviews (IDIs) with 14 key informants. Experienced male researchers (ON and LM) facilitated participatory workshops and conducted IDIs and FGDs with the help of trained female youth researchers (TB and TC).

Participatory workshops, individual interviews and FGDs explored socio-cultural influences on adolescent growth, adolescent sexual behaviour and SRH, relationships, household task and resource allocation, as well as alcohol and substance use norms. Participatory workshops were guided by a bespoke manual, which included sessions from previous initiatives with young people [14,15,21] and lasted four hours, with several breaks, warmers and some refreshments. Activities encompassed various mixed- and single-sex activities, including drawing daily activity charts, role plays of scenarios, debating and voting for and against statements (S1 File). Informed by a topic guide, IDIs and FGDs lasted approximately 45 minutes and 1.5 hours, respectively. All discussions were held in private spaces within participants’ communities.

**Fig 1:**
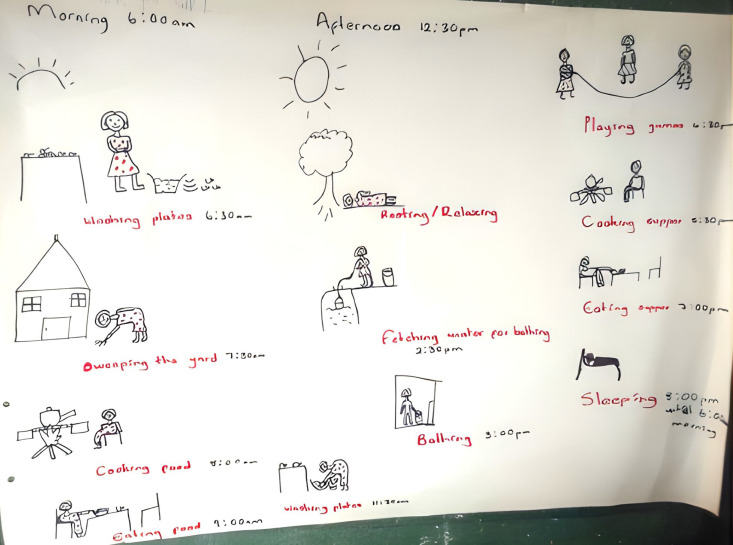
VYA girls activity chart, workshop 1.

All activities/discussions were conducted in Shona, the participants’ language. Workshop outputs were captured on flipcharts (e.g., Fig 1) and/or transferred onto activity-specific electronic forms. With the participants’ permission, workshop discussions, IDIs and FGDs were audio-recorded and later transcribed and translated verbatim into English by TB, TC and LM. More experienced researchers (ON and WM) reviewed all transcripts to confirm their accuracy.

### Qualitative data analysis

A summary was written for each discussion. Fifty percent of each (workshop discussions, IDIs, FGDs) was thematically coded by three researchers (ON, TC and LM), using deductive and inductive approaches. Any discrepancies were resolved through discussion. ON, TC, LM and WM met regularly to develop a common understanding of the codes and their application and to discuss emerging patterns. These deliberations achieved *credibility* (ensuring researchers’ representation of themes fit with participants’ views) and *confirmability* (ensuring interpretations were clearly derived from the data) [22].

Any additional codes identified from this first set of transcripts were added to the coding framework. Transcripts were entered into NVivo 14 (QSR International, Melbourne, Australia) and fully coded using the modified coding framework; care was taken to identify any additional emerging codes. Codes were grouped and emerging themes were identified and where relevant, supported with verbatim quotes.

### Stakeholder workshop

We invited 30 stakeholders to a workshop where we presented formative research findings to: a) validate emerging findings and b) explore how the findings might influence the design of our gender-transformative intervention. Stakeholders were diverse and included VYAs, older adolescents, policy makers, parents/guardians, teachers, traditional/religious leaders, community influencers and community-based organisations representatives. Workshop activities included small group and plenary discussions, which were recorded on flipchart paper, subsequently transcribed, and are summarised below.

### Ethics statement

This study was approved by the national ethics committee, Medical Research Council of Zimbabwe (#2864). Written informed consent was obtained from all participants aged ≥18 years. Assent and parental consent were obtained for those <18 years. Confidentiality assurances were given during the assent/consent process. Names and other personal identifiers were removed from transcripts and other data sources before they were analysed. Participants received US$5 bus fare reimbursements.

## Results

Between April and June 2023, eighty VYAs (50% male) took part in four participatory workshops. All had some primary schooling. However, 37/80 (46%) were out of school. We also held FGDs with 48 older adolescents (50% male) and 24 parents/guardians (50% male) as well as IDIs with 14 key informants. Findings were categorised into the themes that the various activities/discussions explored (i.e., the influence of gender stereotypes on: gender identity, sexual behaviour, SRH, relationships, resource and task allocation and, alcohol/substance use norms). These themes are described in detail below.

### Stereotypes and gender identity

Overall, VYAs were able to distinguish between sex as a biological construct and gender as a socio-cultural construct. For instance, they were able to correctly categorise statements according to whether they were about sex or gender. Statements relating to sex included for example: ‘Boys do not have monthly periods (menstruation), girls do’ and those on gender included: ‘Girls should be gentle; boys should be tough’.

Differing gender stereotypical attributes for boys/men and girls/women, including dress, household tasks and physical build were identified as markers of gender identity. Gender stereotypes also influenced understanding of gender identities. For instance, VYAs generally indicated awareness of the diverse gender groupings in their society. Others, however, identified transgender (*mukosikana -* half boy, half girl) which they confused with homosexuality (a sexual orientation). Of note, terminology for all stereotypes was largely derogatory, owing to societal stigma and influence.

### Stereotypes, SRH and relationship norms

In all four communities, role plays by both boys and girls on ‘What men in your community typically do’, largely portrayed violence (including gender-based violence [GBV]) and multiple concurrent partnering. Of note, within this setting, men’s multiple partnerships are traditionally considered acceptable within certain scenarios (e.g., childless marriage) [15]. Unsurprisingly, both boys and girls seemed to condone boys’ multiple, concurrent relationships while conversely labelling girls with multiple partners or who initiate sexual activity as loose, which testifies to the “sexual double standards” [23] that VYAs are exposed to as they grow up. As one participant put it, *‘If a girl says she wants to sleep [have sex] with you when you have not suggested it yourself, it means she is a prostitute’* (Boy, 14 years).

Boys were deemed to be unable to control their sexual urges; it was their nature to do so *(‘they were just created like that’*) (Girl, 13 years). They were also cast as lacking self-control and portrayed as having an innate determination to fulfil sexual desires (‘*when they want [sex], they really want it’*) (Girl, 14 years). Such portrayals serve to reinforce the gendered local stereotypes that support sexual violence and dominance among boys and men. Consistent with previous findings [13,15], girls/women were expected to display “passive” sexual behaviour (i.e., not expected to initiate any sexual activity or suggest condom use). As one participant remarked, *‘Boys can keep condoms but a girl cannot say to a guy, “I already have a condom”. If she says so, it means that she sleeps around’* (Boy, 12 years).

Menstrual stigma, myths and misconceptions were pervasive among all participating groups. During this time, girls were told to avoid certain chores (e.g., slaughtering chicken, serving in church) as they were deemed “unclean”. *‘All girls in our family know that they should not slaughter a chicken when they are menstruating. Our mother always reminds us’* (Girl, 14 years). Myths related to menstruation were common among boys, including the belief that potential health “effects” of having unprotected sex with a menstruating girl could be prevented by drinking a solution made from mature soot. *‘I heard from my older brother that if you have sex with a menstruating girl, you should put mature soot in a cup of water and drink. Otherwise, your testes will swell’* (Boy, 11 years). The supposed impurity of girls/women during their menses prompted boys to tease girls when they bled on their school uniforms, resulting in some girls missing school.

### Stereotypes and resource, task allocation

Gender stereotypes were also evident in beliefs and norms around resource and task allocation. For example, VYAs were presented with a nuanced vignette of an under-resourced family that had to make the difficult decision of keeping only one of their twins (boy, Edson and girl, Esther) in school. Even when the girl was presented as performing exceptionally well, some participants voted for the boy to stay in school. Their reasoning was that the boy would benefit his family in future, but the girl would get married and benefit a different family instead, or could even fall pregnant before completing her studies. *‘Esther may fall pregnant before finishing school and so, the family will lose out’* (Girl, 13 years). Interestingly, these views were stronger among girls than boys, and likely reflect the high teenage pregnancies within the study communities.

Gender stereotypical beliefs were also evident in household task allocation, as evidenced by gender-specific daily activity charts (see example in Table 1). VYAs made a distinction between ‘masculine’ and ‘feminine’ household chores, with tasks such as sweeping the yard, washing utensils and fetching water considered feminine. Overall, both younger and older adolescents felt that construction and other physically taxing jobs (e.g., water well excavation, prevalent within the community) were a prerogative for boys/men. They could only be performed by girls/women in exceptional circumstances. *‘The tough jobs are only done by women who have lost their husbands or whose husbands have been crippled’* (Boy, 10 years).

**Table 1:** Typical example of daily activity charts compiled by girls and boys separately, workshop 2.

Time	Activity (girls)	Time	Activity (boys)
7.30 am	Sweeping yard	8.00 am-12.30 pm	Playing football
8.00 am	Cooking food	1.00-2.00 pm	Eating lunch
9.00 am	Eating food	2.00-3.00 pm	Watching television
11.30 am	Washing plates	3.00-3.45 pm	Bathing
12.30 pm	Resting/relaxing	3.45-4.30 pm	Watering the garden
2.30 pm	Fetching water	4.30-5.30 pm	Chatting with girls
3.00 pm	Bathing	5.30-6.30 pm	Roaming around
3.30 pm	Playing games	6.30-7.30 pm	Splitting firewood
5.30 pm	Cooking supper	7.30-8.30 pm	Eating supper
7.00 pm	Eating supper		
8.00 pm-6.00 am	Sleeping	8.30 pm-7 am	Sleeping

A common feature of the daily activity charts was that boys had almost twice as much leisure time (∼8 hours) as girls (∼4 hours), as the latter are expected to perform most household chores. During discussions, older women affirmed this societal expectation stating for example that a married woman is expected to *‘Do dishes, clean the house, cook, do laundry for her husband’* (Female guardian, FGD 1).

Additionally, daily activity charts highlighted boys’ greater freedom as they are able/permitted to roam around. When asked whether they wished they were a boy, VYA girls stated among other reasons that it was because they would be able to roam around freely. Parents corroborated this limited freedom when they stated that a parent’s role included ensuring the girl child was home early. *‘To make sure that she [girl child] is home on time, let’s say at 5 [pm]. So, if our daughter is not at home by that time, he [father] has to ask her, “Where are you coming from? What were you doing?”’* (Mother/female guardian, FGD 2). Enforcement of stricter timetables aligns with the gender stereotypical perception that girls are more vulnerable than boys.

### Stereotypes and alcohol/substance use norms

In all four communities, role plays by both boys and girls portrayed alcohol and/or drug use. Overall, VYA girls mentioned that if they were boys, they would refrain from taking alcohol/drugs. Implicit in these activities/assertions was that alcohol and/or drug use was a community-wide norm. Of note, both boys and girls disapproved of this norm, and cited examples of community members who had been adversely affected by alcohol and/or drug use. The general feeling, however, was that it was at least acceptable for boys/men to take alcohol/drugs as they were stronger and more resilient than girls/women. Girls/women who took alcohol/drugs were considered both loose and deviant. *‘A girl who drinks alcohol is loose and unsuitable for marriage’ (Boy, 14 years)*.

### Stakeholder workshop contributions

Stakeholders felt that our formative research findings were a true reflection of what transpired in the study communities. Older participants, however, stated that hearing the findings made them realise that they needed to address some of the apparent inequalities between boys and girls. Especially older women were struck by how girls sometimes wished they were boys and perceived that these sentiments were “cries for help”. Overall, stakeholders highlighted the need for our planned intervention to not only focus on VYAs, but also the wider community.

## Discussion

To inform the co-development of a peer-delivered gender-transformative intervention for peri-urban VYAs to promote positive masculinities and SRH, we conducted formative research to determine and prioritise the gender stereotypes and norms to be targeted among this group. VYAs and their significant others outlined dominant gender stereotypes and how they influence beliefs and norms. Broadly, gender stereotypical traits and roles were linked to norms around, SRH, sexual behaviour and relationships, household resource and task allocation and, alcohol and/or substance use. These data were presented to a group of diverse stakeholders who, together with the researchers, explored how the results might inform the design of our planned gender-transformative intervention.

Overall, VYAs could not distinguish between gender and sexual orientation. In addition, they used derogatory terminology when referring to certain genders and sexual orientations, a result of societal stigma and influence. Efforts to address these negative norms will require a multi-level approach targeting the individual, interpersonal and community levels, as provided for by the socioecological framework [24,25]. This framework emphasises multiple levels of influence and supports the idea that behaviours are influenced by various contexts [24,25], and has been successfully used to tackle other forms of stigma in this setting, including HIV-related stigma [25,26].

Several norms directly linked to SRH were identified, including the belief that boys/men cannot control their sexual desires, can have multiple, concurrent sexual partners and can have forced sex with girls. Consistent with findings from other settings [7], even before VYAs engage in sexual activity, they internalise different “social rules” about acceptable heterosexual romantic engagement for boys versus girls and these differentials affect power dynamics between partners. These pervasive gender norms and behaviours, coupled with suboptimal knowledge of SRHR, are the key drivers of sexual and GBV; the latter is a key driver of the HIV epidemic [27].

Role plays on multiple, concurrent sexual partnerships mirrored community-wide practices. Initiatives to promote positive masculinities will need to highlight that whilst these partnerships are a traditionally acceptable norm, they often result in sexual networks, which have a high risk of especially HIV transmission and/or acquisition [28]. Of course, traditional positive norms such as those relating to men providing for and protecting families and communities, need to continue to be promoted; this is desirable within this setting.

It will also be critical to target boys (and girls) with menstrual health education to address menstrual-related myths and stigma. A pilot study conducted in Uganda tested a multicomponent menstrual health and hygiene intervention that targeted both boys and girls. Of note, a drama skit on menstruation was very popular with both, and was able to engage boys in this topic and de-stigmatise menstruation [29].

Our study highlighted the influence of gender stereotypes on resource allocation. In Zimbabwe and other sub-Saharan settings, these stereotypes and norms have resulted in gender disparities, with the gender gap in education persisting as girls and women receive fewer financial resources [30]. Similarly, although clinics are deemed more accessible to women and girls, there exists a wide gender gap in access to health resources, with women and girls receiving less resources to cater for their health care needs [31]. Barring social desirability bias (the tendency to provide responses thought to be more favourable/acceptable as opposed to being reflective of true thoughts/feelings) [32], the fact that more boys than girls felt that given limited resources, a girl child should be prioritised for education suggests that VYAs’ stereotypes and norms may be amenable to change.

Through the formative research and stakeholder workshop, we were able to identify VYAs’ key influencers, as well as their role in the formation and reinforcement of gender stereotypes and norms. These include the community at large, family members, traditional/religious leaders and certain institutions (e.g., church). It will be important to work across the socioecological framework since individuals are embedded within larger social systems and multiple levels of influence not only exist but interact and are reinforcing [26,33]. Additionally, it will be important to identify and explore the community resources and structures available to ensure sustainability and scalability.

The neighbourhood normative environment (the gamut of norms in each community or neighbourhood) influences how VYAs view (and relate to) alcohol and/or substance use. In their role plays, VYAs depicted various alcohol-inspired negative behaviours, likely reflecting community-wide norms. Of note, VYAs disapproved of both alcohol and/or substance use norms and allied behaviours. To maintain and sustain this positive perception, it will be important to harness the influence of community role models in both denouncing alcohol and/or substance use while also promoting positive and healthy masculinities. In Zimbabwe, role models have been successfully used to promote other health-related interventions (e.g., voluntary medical male circumcision) [34,35].

Data presented here contributes towards one of the few gender-transformative initiatives targeting VYA boys, and therefore contributes to this area’s scant literature. Similar to previous studies [6], our research identified issues related to GBV. However, it additionally identified nuanced issues on norms relating to sexual behaviour, SRH and relationships, household resource and task allocation and, alcohol and/or substance use. These findings, together with inputs from the stakeholder workshop, will inform our prototype intervention. As we iteratively pilot the prototype intervention, we will be able to track any changes in these deeply seated norms. We are however, encouraged by the well-recognised fact that intervening in early adolescence, when attitudes and behaviours are still malleable, provides the opportunity to promote gender-equitable identities, and challenge inequitable gender stereotypes that are harmful to both girls and boys before they are solidified and become less amenable to change [9]. Additionally, our formative research pointed to some norms that were potentially easy to change, which is indicative of the likelihood of attaining positive effects.

Our study adds to the growing body of literature on African masculinities, and the need to understand them through an intersectional lens in relation to other factors [12]. We describe how, among adolescents in impoverished areas, these masculinities are shaped by various social and economic factors including poverty, unemployment and various social vices. Data were gathered through a relatively big sample, which enhances the findings reliability. Additionally, triangulation of methods ensured rigour. Stakeholders felt that findings were a true reflection of study communities’ occurrences; this is an affirmation of the data’s validity. Of note, the use of participatory methods likely minimised social desirability [32], as participants largely brought out their true thoughts and beliefs through say role plays without consciously thinking that they could possibly be judged.

A potential limitation is that our sample was largely drawn from peri-urban communities, which may have different norms and traits than those in other settings; findings may therefore not be representative of VYAs in other parts of Zimbabwe – but the fact that norms were similar to those in other settings in Africa suggests that they may be more broadly applicable. Additionally, most of the issues reported here relate to heterosexual boys. Future research should explore similar issues among VYAs from other settings and other categories of boys (e.g., gender non-conforming boys).

## Conclusions

Our formative research unravelled important insights into gender stereotypes and norms that VYAs grow into. Given their ubiquitous nature, these stereotypes and norms will need to be systematically targeted to influence positive masculinities and SRH among VYAs. While it will directly benefit male VYAs, females also stand to benefit from our proposed intervention as most actions associated with hegemonic masculinities negatively impact their lives. Despite stereotypes and norms being deeply seated, our research suggested that windows of opportunity to influence positive masculinities and SRH existed, and should be ridden on.

## Supporting information

S1 File Participatory workshops manual. (DOCX)
