# Supplementary material for: Exploring gender stereotypes and norms among peri-urban very young adolescents in Zimbabwe using participatory and qualitative approaches
Source: PLOS Glob Public Health. 2025 May 29;5(5):e0003845. doi: 10.1371/journal.pgph.0003845 (PMC12121727; doi:10.1371/journal.pgph.0003845)
Supplement: S1 File — (DOCX) [file pgph.0003845.s001.docx]

**Participatory Research with Very Young Adolescents**

The Research to promote positive masculinities among very young adolescents (VYAs, 10-14 years) in Zimbabwe initiative works with boys, in addition to girls through gender-transformative (GT) programming, to challenge gender inequalities and harmful norms related to masculinity.

The research is operational in 4 marginal communities in Harare, Zimbabwe and aims to engage VYAs. Many of the attitudes and values targeted by the intervention are culturally based and as such may not be appropriately captured by standard measures. We therefore developed a participatory research framework which allows VYAs to define their own attitudes and norms around issues (at least to some extent) and the research team to record these both qualitatively and quantitatively.

***Aims of the participatory research workshops***

- To use participatory research workshops (PRWs) to assess gender norms among VYAs and how they influence adolescent SRHR.
- To quantify (e.g., through counting, coding or ranking) qualitative responses, where appropriate.
- To use these data to co-develop an intervention with VYA.

The workshop is designed to be run with 20 to 30 people over a day. It is divided into 5 sections – an introductory section (A) and then four sections assessing norms and values relating to adolescence and gender roles (B), gender norms and adolescent relations (C), and gender norms labour and resource allocation.

The workshop is run by four facilitators. Where possible the plenary discussions are tape-recorded. Each of the facilitators takes notes throughout the sessions, taking down direct quotes of participants where possible/appropriate. Data collected onto flip charts, maps, into ballot boxes etc. are transcribed onto specially designed data collection forms.

***Participatory techniques***

The workshops are designed to be as participatory as possible. Each activity was also designed to maximise the possibility of quantifying the data that emerges.

The following techniques are used in this module:

- Mapping
- Plenary
- Group presentations
- Voting
- Picture codes
- Games
- Role play
- Story telling
- Debate

***Instructions for workshop facilitators***

This SOP sets out in detail exactly how the workshop as a whole and each activity should be run. The workshop facilitators need to properly familiarise themselves with the materials prior to running a workshop.

***Planning***

Before each workshop, facilitators need to decide which facilitator is going to run each activity. The note taking duties and tape-recording duties need to be assigned. All the materials that are required for the workshop need to be assembled.

Although each activity is already planned in detail, the facilitator needs to properly facilitate it for it to work at its best. For each activity, the facilitators need to think about exactly how the activity will be introduced and how it will be rounded off. Some activities will cause lively debate and others require people to reflect in private. How an activity is introduced will affect the mood and motivation of participants to fully engage. Equally important is how an activity is rounded off so that emotions and ideas are not carried over from one activity to the next.

***Preparing the venue***

Facilitators should arrive at the venue at least 20 minutes before participants arrive. Sessions can take place in or outdoors. The venue should be clean and have enough space for participants to meet in plenary as well as in small groups. There should also be somewhere to hang flipcharts.

Sitting (mats or benches) should be arranged in a semicircle. Water should be available for refreshment.

***Standardisation of the workshop framework:***

To be able to compare data from different communities it is important that activities are run in a standardised way. Each activity should be run exactly as outlined in this SOP. If, at any time, it becomes necessary to alter the structure / course of an activity from that outlined, it is important that this change is recorded so that it can be considered when conducting the analysis.

Remember in each activity that we are trying to get participants to think about the situation in their lives – either in their community, their household, or their own personal situation. Participants need to be reminded of this whenever they appear to be getting off track.

***Note-taking/Record keeping***

Each community in which the workshop is run will have a Community Code. Each group with which the activity is run will have a group code (group ID) and each activity has a code (activity ID). All paper generated as part of the workshop should be appropriately labelled as detailed in the SOP to ensure that it can be identified later.

Each facilitator should have their own notebook and record any details that they consider of interest either as direct quotes (“”) or as notes.

In addition, each facilitator should take notes on specific aspects of each activity – indicated throughout the SOP by the following symbols 🖎 and ▽.

***Additional points:***

- Facilitator notes for each activity should start on a new page. At the top of each page, include the Community Code, group ID, activity ID, date, and page X of Y.
- Do not record notes on the back of pages.
- When recording summary statements or direct quotes from participants, record the participant gender in the margin before each comment or summary statement. If the data is a direct quote, place it in quotes. If the data is a summary statement, do NOT place it in quotes.
- Notes should include quotes that the facilitator considers representative or different from the stated views. Also quotes will include data that provides additional insight or context to the data being recorded during the activity.

***Completing the data collection forms***

For some of the activities, the facilitators are required to transcribe data from the flip charts onto forms or transcribe data from their notes in Shona into English. When the facilitator is required to do this, it is marked in the SOP by a ▽ symbol. The form for completion will be identified.

***Materials to take from the office.***

Materials required for each workshop are listed at the beginning of each activity

***Information for the facilitator***

***Objectives***

By the end of this session, participants will:

- Understand the purpose of the participatory workshop
- Have signed a consent form
- Have provided demographic data
- Established ground rules
- Introduce everyone

***Materials***

- Lever Arch file for the consent forms (blank consent forms inside)
- Blank Consent forms (2 per participant)
- Pens
- Ball (for the introductory name game)
- Flip chart paper, markers, sticky stuff, sticky notes (different colours)

***Introduction***

1. As participants arrive, greet them, and let them know that we will wait to get started until everyone has arrived.
2. Once everyone is settled, welcome and greet everyone and thank them for coming.
3. Ask participants what they know about the programme and let them call out ideas.
4. Then, ensure that you explain the following:

- The Research to promote positive masculinity project is exploring positive masculinities and sexual health among VYA.
- The workshop today is trying to better understand the lives of VYA in their community. Trying to understand what it is like to be a young person in this community.
- Workshop will take place over one day. Will run a series of different activities to better understand what it is like to be a very young adolescent in this community.
- Workshop is best when everyone participates and contributes.
- Let them know a bit about yourselves:

1. you are researchers working on the project;
2. have been studying adolescent SRH issues for the last few years;
3. have explored this in several ways:

- Focus Group Discussions
- In depth interviews
- Surveys

Be sure to set a participatory mood. Try not to talk the whole time but where a chance arises, allow participants to contribute.

***Consent***

Explain the following:

1. Before we begin, for you to participate, we will need you to sign a consent form that shows that you are

- understand the purpose of the workshop and
- that you are willing to participate.

1. Hand out a blank consent form to each participant. Explain that you are going to read out the information sheet on the back of the form to everyone.
2. After you have done this, ask if there are any questions as a group. Answer any questions that arise.
3. Ask one person from the group to summarise what they think is happening over the duration of the workshop. Ask if there are any additional contributions to the summary. The participants’ explanation should help you to ensure that they are well informed of the purpose of the workshop and the consent process.
4. Their summary should include the following points:

- Anonymous – no names being collected.
- Participatory activities
- By signing the form, they agree to participate actively over the duration of the workshop.

1. Have participants line up with those that are ready at the front and those that want to read it themselves at the back. Each facilitator takes each participant one at a time and has them complete the consent form.
2. Ask them each question listed on consent form. Tick each box as you complete each question.

- Print participant’s name, and the date.
- Print your name, and the date
- Have participant sign. Then the facilitator signs.

Remember to complete 2 (two) consent forms for each participant. Keep one form and file it in the lever arch file. Hand the other completed consent form to the participant. Let them keep the pen they used, reminding them that there will be times when they will use it over the course of the workshop.

Have one facilitator file all consent forms in the Lever Archer File.

***Complete the Participant Demographic Form***

1. After the participant has consented complete the Participant Demographic Form.
2. This can be done in a participatory way. Ask participants who are 10 to stand up. Count boys and girls. Do this for ages 11, 12, 13, and 14. Write these down on the Participant Demographic Form.

**Introducing Participants (10 Minutes)**

**Objective:** To enable all participants to know each other better.

**Methods:** Individual or small group introductions

**Aids**: Flip charts, markers, soft ball,

**Procedure:**

Participants can introduce themselves in a lively and engaging and memorable way. Different methods can be used for introductions as follows:

- **Catch the Ball**

The facilitator gathers participants in a circle and randomly invites one participant to the centre, gives them a soft ball. Subsequently, the participant introduces themself and randomly throws the ball to the next participant and takes their place in the circle until all participants have introduced themselves. To make it interesting, each participant can share their favourite colour/ fruit/ sport/ musician/hobby.

- **Introducing Your Neighbour/Meet my partner.**

All participants will be asked to randomly select a partner/‘neighbour’ (preferably someone they have never met before). The participants should be given time with their partner to get to know each other gathering information such as their partner’s name (and preferred name), where they come from and their favourite colour/ fruit/ sport/ musician/hobby. Afterwards, each participant will be given at least one minute to introduce his or her partner to the entire group.

- **Self-introductions**

All participants will be given a chance to introduce themselves, providing vital information such as their name (and preferred name), where they come from and their favourite colour/ fruit/ sport/ musician/hobby.

🖎Facilitators can (with participants’ consent) write names/nicknames of participants on flipcharts which will work as a memory aide for all participants.

**Ground Rules: (5 Minutes)**

**Objective:** To develop ground rules to improve group interaction, prevent/reduce misunderstanding/disagreements and to make meeting more productive and enjoyable.

**Method:** All participants

**Aids:** Flipcharts, markers

**Procedure:**

1. Whilst standing in a circle, explain that for the exercises to be successful and enjoyable we should set down some ground rules that we all follow. Ask everyone to think of two things that they think are important for this workshop to run smoothly.
2. Go around the circle asking each person to say out their rules. Once they have spoken, they can sit down.
3. Write each rule on a flip chart.
   1. If they are inappropriate (e.g., every person should be forced to attend the meetings) explain why they are not suitable. Keep going until everyone has sat down or all the rules have been exhausted. If someone’s ideas have already been said when their turn comes they can sit down and go on to the next person.
   2. If there is a debate, or the issue seems divisive in some way, ask the group to vote on it before including it on the chart.
   3. Facilitators can create a ‘PARKING LOT’ in the form of a flipchart where contentious issues are ‘parked’ for later discussion.
4. Ensure the following rules have been covered (add your own if necessary and ask the group what they think to try and get support). This can be done while the next activity has begun or during a break. If you see that something is missing, you can add it at the beginning of the next activity, stating, ‘I notice that we haven’t mentioned ….’

Avoid arguments, instead discuss differences in opinion until you have reached a conclusion.

- Everyone should participate
- Respect each member of the group
- Listen to what each person has to say
- Be open and honest – say what you really believe rather than what is the “right thing”
- Encourage those who are shy to participate
- Keep confidentiality – if people in the group talk about personal things don’t gossip about them with others
- Keep to the subject of discussion - do not get distracted with other topics
- Be open minded to other points of view – try not to judge people
- Don’t fear what others will think of your contribution
- Be clear about what you want to say
- Be on time both days to benefit from the whole workshop

**🖎 Facilitators Note:** Refer to the ground rules whenever it is necessary to help bring the group back together.

**Housekeeping (5 Minutes)**

Before getting into the finer details of the workshop, it is also essential to set out and clarify housekeeping issues**.** Essential housekeeping issues to consider could include:

- Starting, break times and finish time
- Etiquette about phone usage (Switching off/silencing cell phones)
- Location of bathroom facilities
- Refreshments and etiquette (If refreshments are being served, inform participants to either wait for breaks or to help themselves)

Thank everyone for their suggestions and participation. Introduce the first activity.

**Section A - Adolescence and Gender Roles**

**Activity A1: Assessing Knowledge of Gender (20 Minutes)**

**Objective:** To assess if participants know the meaning of gender **Methods:** Small groups, role play, Plenary

**Aids:** Flipcharts, markers

**Procedure:**

1. Divide the participants into small groups of not less than 5 participants but not more than 10 participants (depending on the size of the whole group)

2. Ask the small groups to deliberate on their understanding of gender and the different types of gender as well as gender conformity.

3. Ask the groups to prepare their feedback in the form of role play in front of the larger group. While small groups will discuss all the questions, share questions for groups to prepare their role play on. The following questions can be used as guidelines.

a) What is gender?

*Chii chaunonzwisisa pamusoro pekuva uye nezvinotarisirwa pakuva munhurume kana kuve munhukadzi*

b) How do young children learn their gender? (Role play)

*Vana vanodzidza sei pamusoro pehusikana/hunhukadzi kana hukomana/hunhurume hwavo*

c) How many genders are there?

*Kune mhando ngani dzingawanikwe pakuve murume, mukadzi kana zvimwewo?*

d) How do men show their manliness in society? (Role play showing dominant and alternative forms of being men).

**Note to facilitators: Participants to switch roles after own-gender role play (d &e)**

*Vanhurume vanoratidza sei hunhurume hwavo munharaunda?*

e) How do women show that they are women in society? (Role play showing dominant and alternative forms of being women)

*Vanhukadzi vanoratidza sei kuti vanhukadzi/hunhukadzi hwavo munharaunda?*

4. Reconvene the groups and ask them to perform their respective role plays and ask the others to describe what they are observing and what it means to them?

**🖎Facilitator: record responses onto General Transcription Form A1T**

**Activity A1a: GENDER GAME: Differences Between Gender and Sex (20 Minutes)**

**Objective:** To see if participants can distinguish gender from sex. **Methods: Small groups, Plenary**

**Aids: Flipcharts, markers, blank gender/sex forms**

**Procedure:**

1. Divide the participants into small **GENDER and AGE specific groups** of not less than 5 participants but not more than 10 participants (depending on the size of the whole group)
2. Ask each group to discuss and fill in their answers on the differences between gender and sex. Respondents’ gender to be indicated on forms.
3. Invite group representatives from one group to present their completed form explaining their choice of responses.
4. Initiate discussion with other groups if they put a response different from presenting group.

| **STATEMENT** | **ANSWERS** | |
| --- | --- | --- |
|  | **GENDER** | **SEX** |
| Women give birth to babies, men don’t.  *Vakadzi vanotakura pamuviri nekuzvara vana, varume havatakuri pamuviri* |  |  |
| Girls should be gentle; boys should be tough.  *Vasikana vanofanira kupfava/kuninipa, vakomana vanofanira kuwoma* |  |  |
| Boys can stay out late, girls cannot.  *Vakomana vanogona kunonoka kudzoka kumba, vasikana havakwanisi* |  |  |
| Boys do not have monthly periods (menstruation), girls do  *Vakomana havaendi kumwedzi, vasikana vanoenda kumwedzi* |  |  |
| Girls should wash dishes and cook, boys cannot.  *Vasikana vanofanira kusuka ndiro nekubika, vakomana havafaniri* |  |  |
| Boys can have many girlfriends, girls cannot.  *Vakomana vanokwanisa kudanana nevasikana vakawanda, vasikana havakwanisi* |  |  |
| Girls and boys develop breasts in adolescence.  *Vasikana nevakoma vanomera mazamu panguva yekuyaruka* |  |  |

**🖎Facilitator:** Record responses on flipcharts and transcribe responses to Gender-Sex Score Card **A1a**

**Activity A2: What is Adolescence? (20 Minutes)**

**Objective:** Participants should be able to describe adolescence and exciting or challenging things about it.

**Methods:** Small groups, role play, Plenary

**Aids:** Flipcharts, markers

**Procedure:**

Divide the participants into 2 gender specific groups of not less than 5 participants but not more than 10 participants (depending on the size of the whole group)

1. Ask participants to reflect on (and write on a flipchart **A2F** and **A2M**) what they understand adolescence means. The following points should come out:

Adolescence is:

Kuyaruka:

- Period between childhood and adulthood
- *Inguva iri pakati pehupwere nekubvazera/kuyaruka*
- Period of physical, social, and emotional change
- Inguva yekuchinja kwehengo dzemuviri, kuwadzana nevamwe, nemanzwiro/mafungiro
- Period of sexual development
- *Inguva yekuchinja kwemanzwiro nemafungiro panyaya chekuita nezvepabonde*
- Period of discovering who you are and what is important to you.
- *Inguva yekudzidza kuti ndiri ani uye chii chakandikoshera*
- Period of learning
- *Inguva yekudzidza*

2. **Ask participants:**

1. What challenges young people face during adolescence?

*Ndeapi matambudziko anosanganikwa nawo nevechidiki mukuyaruka*

1. What is exciting during adolescence?

*Ndezvipi zvinonakidza/zvinofadza panguva yekuyaruka*

🖎 Facilitator transfer responses from flipcharts to General Transcription Form **A2FT A2MT**

**Activity A3: Gender Socialization & Gender Roles (20 minutes)**

**Objective:** To ascertain VYA’s attitudes around different gender roles

**Methods: Small groups, role play**

**Aids: Flipcharts, markers**

**Procedure:**

1. As a group, ask them to complete the following:
2. I know I am a boy/girl because …….

*Ndinoziva ndiri* *mukomana/musikana nekuti….*

1. I am glad that I am a boy/girl because ……

*Ndinofara kuva mukomana/musikana nekuti….*

1. I know I am not a boy/girl because ……

*Ndinoziva handisi mukomana/musikana nekuti….*

1. If I was a boy/girl, I could ……

*Dai ndiri mukomana/musikana ndaizokwanisa ku…*

1. If I was a boy/girl, I could not …..

*Dai ndiri mukomana/musikana ndaisazokwanisa ku…*

🖎**Facilitator** Allocate flipcharts to groups and list responses to Question 1. Label flipcharts **A3F** for girls and **A3M** for boys. Responses to be transcribe to General Transcription Forms **A3FT A3MT**

**Activity A4: Gender Identities & Stereotypes (20 Minutes**

**Objective:** To understand how gender norms stereotypes influence gender identities

**Methods:** Small groups, role play, plenary

**Aids:** Flipcharts, markers

**Procedure**

1. Ask the young participants to give their responses quickly to the following phrases one by one as you move around the room.

- “Girls don’t like boys who…”
- *Vasikana havafariri vakomana vano….*
- “Girls don’t like girls who…”
- *Vasikana havafariri vasikana vano….*
- “Boys don’t like girls who…”
- *Vakomana havafariri vasikana vano….*
- “Boys don’t like boys who…”
- *Vakomana havafariri vakomana vano….*

1. After each phrase, discuss the similarities and differences in the responses.

**🖎Facilitator:** Record responses on a flipchart dividing responses into **A4F** for girls and **A4M** for boys. Responses to be transcribed to General Transcription Form **A4FT** and **A4MT** respectively.

**Activity A5: Act Like a Boy/ Act Like a Girl (20 minutes)**

**Objective:** Examine how cultural messages about gender can affect human behaviour in boys and girls

**Methods:** Plenary session, small gender-specific groups

**Materials:** flip charts, markers

**Procedure**

1. Ask the following questions:
2. What emotions are girls or boys not allowed to express?

*Ndeapi manzwiro asingabvumidzwi kuti vasikana/ vakomana vabudise pachena?*

1. Can girls/women or boys/men live outside the box? Is it possible for girls/women or boys/men to challenge and change existing gender roles?

*Vasikana/vakadzi kana vakomana/varume vanokwanisa here kurarama kunze* *kwezvinotarisirwa? Zvinokwanisika here kuti vasikana/vakadzi kana vakomana/varume vapikise uye vachinje* *zvinotarisirwa pakuva musikana/mukadzi kana mukomana/murume?*

1. What are the consequences of acting outside the box?

*Ndezvipi zvinowira munhu anenge aita zviri kunze kwezvinotarisirwa*

1. Is it different for boys and girls in rural vs. urban areas?

*Zvinosiyana here kune vakomana nevasikana vari kumamisha zvichienzaniswa nevari kumadhorobha?*

1. When is it OK for a girl/woman or boy/man to live outside the box?

*Ndepapi pangabvumirwa musikana/mukadzi kana kuti mukomana kurarama kunze kwezvinotarisirwa*

🖎Facilitator Note responses to this discussion on a flipchart and transcribe to General Transcription Form **A5FT** for girls and **A5MT** for boys.

**Warmer: (make it somewhat active)**

**Ask a participant to volunteer to lead a group song or dance that gets everyone up and moving around (5 Minutes)**

**Section B – Gender Norms & Adolescent Relations**

**Activity B1: What is love? (20 Minutes)**

**Objective:** To assess the influence of gender norms on love/relationships

**Methods:** Small groups, plenary

**Materials**: Flip chart paper, markers, sticky stuff

**Process:**

1. Divide the group into 2 single-gender groups. Ask
2. ‘What is love?

Rudo chii?

Probe:

1. How do you show you love someone?

Unoratidza sei kuti unoda mumwe munhu

1. How do you know if someone loves you?

Unozviona/unozviziva sei kuti mumwe munhu anokuda

1. Write their responses on the flip chart. Have the group brainstorm all their responses to this question. Facilitator records all statements onto a piece of flip chart paper. Code this **B1F**and **B1M** respectively

▽ These comments will be transcribed onto a General Transcription Form. Label it **B1TF/B1TM**.

1. After they have created their statements, bring the two groups back together and have them present their statements to the other group. Thank them for their work.

**Activity B2: Perceptions around Sexuality (10 minutes)**

**Objective:**

**Materials**

- Flip chart paper, markers, sticky stuff
- YES/NO cards
- FORM B2

*Advance Preparation:* Have two pieces of flip chart paper. On one, write the women’s statement (code it **B2F**), and on the other, write the men’s (code it **B2M**).

**Forms required:**

*During the activity:* ***Form B2A***

**Process**

1. Split participants into single-gender groups.

**Ask girls to discuss–**

F. ‘Do girls/women have sexual desires?

Vasikana/vakadzi vane havi yebonde here?

Then, ask:

Do girls/women *feel free to show* their sexual desire?

Vasikana/vakadzi vakasununguka here kuratidza havi yavo yebonde?

(Probe: If not, why not? If so, how do they do this?’)

(Bvunzurudza: kana vasingakwanisi, sei vasingakwanisi? Kana vachikwanisa, vanokwanisa sei kuzviita?)

**Ask boys to discuss –**

M. ‘Are boys/men able to control their sexual desire?

Vakomana/varume vanokwanisa here kukurira havi yavo yebonde?

(Probe: If not, why not? If so, how do they do this?’)

(Bvunzurudza: kana vasingakwanisi, sei vasingakwanisi? Kana vachikwanisa, vanokwanisa sei kuzviita?)

Then ask:

Boys/men use force when they are unable to control their sexual desire?’

Vakomana/varume vanoshandisa chisimba kana vatadza kukurira havi yavo yebonde?

1. Have the facilitator write down a summary of each point made under the statement on the flip chart. Code each flip chart as **B2F** and **B2M** respectively.
2. After their discussion, and while still in single gender groups, have participants vote if they agree or disagree with both their statement and the statement from the other group.

**NB: Have each group answer both sets of questions**

- 1. Place YES/NO cards in different places and have participants go and stand next to their vote.

🖎Record the vote on **VOTING SHEET FORM B2** and transcribe statements from **B2F** and **B2M** onto **B2FT** and **B2MT**

**Activity B3: Gender violence – Boy hitting Girl (15 minutes)**

**Objective:**

**Materials:**

- Picture code **B3** Boy hitting a Girl
- Ballot box marked **B3**
- Pre-printed ballot sheets. Count out correct number for girls (G) and boys (B).

**Forms required:**

After the workshop: Form **B**, under **B3**

Note: Back at the office when ballot box is opened, we need to write Community Code on the back of each piece of paper.

**Procedure:**

1. Ensure that you have one big group. Ask,

‘What does this picture show?’

‘Mufananidzo uyu unoratidzei?’

1. Allow participants to describe the picture. Then ask,

‘Why might this be happening?’

Chii chingadero chaita kuti izvi zviitike?

🖎 Record responses in notebook under ‘**B3**’.

This will get translated and transcribed onto the General Transcription Form coded as **B3T**

∇

1. Place the ballot box in front of the group.
2. Give each participant a small piece of paper, the young girls should have **PINK** stick notes and the young boys should have **YELLOW**. Explain that you are going to read out 2 statements. For each statement you are going to give them a piece of paper on which to place their vote. Show that the paper has a box next to the word *Hongu* and next to the word *Kwete*. Ask them to put a **✓** for **Hongu/Yes** **🗶** for **Kwete/NO** in the box that is their answer to the question.
3. Read first statement:

‘Number 1. If a wife / girlfriend does something wrong, she should expect her husband / boyfriend to punish her.’

Kana mukadzi / musikana aita chimwe chinhu chakaipa anofanira kutarisira murume / mukomana wake kuti amurange’

1. Have everyone place their paper in the ballot box. Hand out the second piece of paper and explain that you are now going to read out the second statement:

‘Number 2. ‘It is never okay for a man to hit his girlfriend or his wife.’

. ‘Hazvina kunaka zvachose kuti murume arove musikana kana mukadzi wake.’

1. Have everyone fold their piece of paper so their responses are inside the fold and ask them to come forward and place it in the locked ballot boxes.

- Have the group discuss these ideas generally after they have privately voted.
- 🖎On return to the office the ballot boxes will be opened, and the votes counted with the numbers recorded on Form **B** under **B3** for each of the separate questions.

**Activity B4: Gender violence – Boy hitting Girl (15 minutes)**

**Objective:**

**Warmer: make it somewhat active**

**Ask participant volunteer to lead a group song or dance that gets everyone up and moving around**

**Section C Gender Norms, Labour & Resources Allocation**

**Activity C1: Education Story (15 minutes)**

**Objective:** To assess how gender norms influence family decisions on resource use

**Methods:** Small groups, Voting

**Materials:** Voting cards: *Edson*, *Esther*, *I don’t know*

Flip chart, magic markers, sticky stuff

**Forms required**

*During the activity:* Form **C**

*After the activity:* Form **C1T**

**Procedure**

1. Facilitator: Read out this story: **(3 minutes**)

Baba and Amai Madanga are subsistence farmers. They have one set of twins aged 16, a boy Edson, and a girl Esther. Both are in Form 3. The previous farming season was a drought. The tough situation has been compounded by an increase in school fees at the secondary school. The family’s coffers only allow them to send one child to school. Esther got excellent marks last term whereas Edson’s marks were poor.

Baba namai Madanga vanorima zvekungozvidyirawo. Vane mapatya ane makore 16, mukomana anonzi Edson nemusikana anonzi Esther. Vose vari muform 3. Mwaka wapera wakange uri wenzara. Kuoma kwezvinhu kwasangana nekukwidzwa kwemari yechikoro yekusekondari. Mhuri iyi ine mari shoma inoita kuti vakwanise kuendesa mwana umwechete kuchikoro. Esther akapasa zvakanyanya term yapfuura uye Edson akafoira.

1. Ask.

‘Who should they choose to remain in school?’

‘Ndeupi mwana wavanofanira kuendesa kuchikoro pavaviri ava? Sei muchidaro?’’

1. Place voting cards ***Edson***, ***Esther***, and ***I don’t know*** in different places. Ask each person to go and stand under the card that represents their vote.
2. Count the vote by gender and record on **Form C** under **C1**
3. Ask each group to say why they are standing where they are.

🖎 Record statements associated with each group. Ensure it is recorded as C1. Divide your lined page into three sections Edson, Esther, and I don’t know. This gets transcribed into English onto General Transcription Form coded as C1T, later.

1. Then ask:

*What about if Edson’s marks were as good as good as Esther’s? Who do you think should go to school now?*

*Ko dai Edson anga akapasawo saEsther? Munofunga kuti vaifanira kuendesa ani kuchikoro ikozvino?*

1. Record this vote again on Form C.

Scale: Assess how much of the discussion revolved around economic security.

Scale: Assess how much of the discussion revolved around patriarchy.

**Activity C2: The 24-hour clock (20 Minutes)**

**Objective:** To understand gender norms influencing gender-based time-use trends

**Methods:** Two groups, Role play

**Aids:** Flipcharts, markers

**Procedure:**

1. On a flipchart, divide the day into several parts, as shown in the table below
2. Divide participants into two gender specific groups between 4 and 10 participants
3. Ask each group to list the task(s) done by girls and boys from morning, afternoon to evening( Include times where possible)
4. Ask the groups to discuss why it is that men and women cannot do a task that is different from their gender assigned task.
5. Ask groups to do some role play on the tasks for men and women and ask the others to comment on the role play. Label the flipchart C2

**Switch for them to map out the other gender’s activities**

**🖎Facilitator:** Note down observations and responses onto flipchart **C2F** and **C2M** and subsequently transcribe to **C2MT** and **C2FT** respectively**.**

**Activity C3: The Reason Why: Drug and Substance Use**

**Objective: To explore norms influencing drug and substance use**

**Materials:** Flipcharts, markers

**Methods:** 4 Small mixed-gender groups, plenary

**Procedure**

1. Inform participants that in this section you are going to be discussing drug and substance use.
2. Proceed to invite participants to list substances/ types of alcohol that are commonly found in their community. Ask for those that are smoked or inhaled and proceed to those that are drunk or ingested. Write the names on a flipchart. Ask them to indicate those taken by VYA.
3. Divide participants into 4 small groups and task each group to brainstorm and list:
4. Reasons why VYA smoke/inhale the listed substances.

*Zvikonzero zvinoita vechidiki vachangoyaruka vasvute zvadomwa izvi*

1. Reasons why people drink/ingest the listed substances.

*Zvikonzero zvinoita vechidiki vachangoyaruka vamwe/vamedze zvadomwa izvi*

NB: Each group must list at least five reasons for their allocated task.

1. After they have listed their reasons, stick up the lists and discuss the reasons given with the entire group adding other reasons that may not have been listed.

NB: Ensure that “peer pressure” is added to the Reasons Why lists and proceed to discuss why peer pressure influence peers’ reasons why they may drink or smoke.

1. Initiate discussion on whether boys may be allowed to drink/smoke and why girls may not be allowed to drink.
2. List gender specific responses arising from this discussion.

▽Record reasons in Notebook and transfer to General Transcription Form **C3.**

**Closure**

Run SRAN.

- Summarize
- Relate back to beginning (introduction)
- Provide them with a sense of achievement.
- Do not give them anything new.

Run a closing game.

Thank the participants for their participation. We have learned a lot. Hope to use this information to better understand the lives of very young adolescents in Zimbabwe.
